# Biting time of day in malaria mosquitoes is modulated by nutritional status

**DOI:** 10.1186/s12936-025-05550-z

**Published:** 2025-09-30

**Authors:** Catherine E. Oke, Samuel S. C. Rund, Maxwell G. Machani, Abdul R. M. Sabtiu, Yaw Akuamoah-Boateng, Yaw A. Afrane, Sarah E. Reece

**Affiliations:** 1 Institute of Ecology and Evolution, School of Biological Sciences, University of Edinburgh, Edinburgh, UK; 2 Institute of Immunology and Infection Research, School of Biological Sciences, University of Edinburgh, Edinburgh, UK; 3 Center for Research Computing, University of Notre Dame, Notre Dame, USA; 4 Department of Biological Sciences, University of Notre Dame, Notre Dame, USA; 5 Entomology Department, Centre for Global Health Research, Kenya Medical Research Institute, Kisumu, Kenya; 6 Department of Medical Microbiology, University of Ghana Medical School, College of Health Sciences, University of Ghana, Accra, Ghana

**Keywords:** Anopheles, Transmission, Vector behaviour, Daily rhythms, Food availability

## Abstract

**Background:**

Transmission of vector-borne pathogens follows daily rhythms, occurring at the time of day that vectors forage for blood. Control measures, such as insecticide-treated bed nets (ITNs), have been particularly successful for reducing malaria transmission because they exploit the nocturnal biting behaviour of the *Anopheles* spp. that vector malaria. However, shifts in biting behaviour to earlier or later hours when people are unprotected can undermine the efficacy of ITNs. Despite the implications for malaria transmission, the mechanisms underlying these shifts remain poorly understood. Because food availability mediates activity and foraging rhythms, and ITNs block access to human blood as a food source, it was hypothesized that nutritional deprivation could cause mosquitoes to shift their biting behaviour to earlier or later in the diel cycle.

**Methods:**

Female *Anopheles gambiae *sensu lato (*s.l.*) mosquitoes were provided with a blood meal on day 3 post-emergence, and access to one of three feeding treatments that varied in nutritional resources: (i) 0.5% sucrose, (ii) 10% sucrose, or (iii) 10% sucrose plus an additional blood meal on day 6. Mosquitoes were released into a semi-field system on day 10 with human-mimic traps to investigate how food availability impacted the time of day that mosquitoes host seek.

**Results:**

Nutritional resources determine both the likelihood and time of day that host-seeking occurs. Specifically, low-resourced mosquitoes were 2–3-fold more likely to host seek overall, and 5–10-fold more likely to host seek at an earlier time of day than well-resourced mosquitoes (fed 10% sucrose with and without an additional blood meal), which predominantly sought a host in the second half of the night time.

**Conclusions:**

This study reveals that mosquito nutritional condition drives plasticity in biting time of day, suggesting it is an underappreciated contributor to residual malaria transmission. Furthermore, the findings suggest that targeting mosquito nutrition (e.g. sugar-baited traps) could influence mosquito behaviour in ways that affect the success of ITNs. More broadly, understanding the drivers of biting time of day variation is crucial for the future success of vector control tools and controlling malaria transmission.

**Supplementary Information:**

The online version contains supplementary material available at 10.1186/s12936-025-05550-z.

## Background

The transmission of vector-borne diseases (VBDs), such as malaria and dengue, relies on the foraging rhythms of insect vectors, which take up pathogens from an infected vertebrate host during a blood meal and transmit them to a new host during a subsequent blood-feeding event [1, 2]. For example, *Plasmodium* parasites, the causative agent of malaria, are vectored between humans by *Anopheles* spp. mosquitoes, which are generally nocturnally active and preferentially bite between the hours of 11 pm and 4 am (*i.e.* the ‘classical’ time window) [3–5]. Insecticide-treated bed nets (ITNs) exploit these rhythmic foraging behaviours, and are consequently one of the most effective methods for curbing the spread of malaria [6, 7], averting 68% of deaths since 2000 [8]. Despite this success, residual transmission occurs, in part due to the evolution of physiological insecticide resistance (e.g. biochemical and morphological modifications) and behavioural resistance in vector populations [9]. In particular, millions of clinical malaria cases are predicted to be occurring annually due to mosquitoes altering the time of day they bite [10].

There are increasing reports that *Anopheles* spp. are shifting their biting time of day to earlier in the evening (‘early’ biting) or later in the morning (‘late’ biting) when humans are unprotected by ITNs [4, 5, 11–15], undermining the success of ITNs especially in high-coverage regions. However, the extent to which mosquitos can shift biting time of day and the range of potential drivers are poorly understood. *Anopheles* spp. biting rhythms are weakly heritable, so shifts in biting time of day are expected to be predominantly driven by phenotypic plasticity [16]. Phenotypic plasticity evolves to enable organisms to alter (via epigenetic mechanisms, for example) aspects of phenotype, including behaviours, in manners that maximize fitness in response to environmental variation [17]. Furthermore, variation in physiological condition and resource limitation can cause animals to alter their foraging rhythms [18] in adaptive (i.e. fitness enhancing) ways [19]. For example, low food availability promotes a shift to diurnal foraging patterns in small nocturnal rodents, which minimises energy loss by remaining in burrows during particularly cold nights [20, 21]. Similarly, mosquitoes may garner greater benefits from shifting biting time of day when they are in poor nutritional condition, because the fitness benefits associated with acquiring a blood meal at suboptimal times of day outweigh the increased risks of desiccation and predation. Such context-dependent variation in the costs and benefits of plastic shifts in biting time may explain why biting times have seemingly not significantly changed in some *Anopheles* spp. populations despite high ITN use [22, 23], and why the evolution of behavioural resistance varies across populations in manners that correlate with the abundance of sources of nutrition for mosquitoes [24].

Mosquitoes rely on blood and sugar meals for reproduction and survival, with female mosquitoes utilising proteins in blood for egg production, and using sugar sources to accumulate energy reserves for powering energetically demanding processes, such as flight [25]. Their feeding ecology is under circadian clock control [26], with females seeking blood predominantly at night and approximately every 3 days to fuel gonotrophic cycles of egg development and oviposition [27], and feeding on sugar sources in between these cycles to promote survival until future biting opportunities [28, 29]. Mosquitoes are likely to vary in their nutritional status because the type and abundance of sugar sources available to mosquitoes varies across habitats and seasons [25, 30], and vector control tools such as ITNs can limit access to protein rich blood meals [31]. In response to variation in food sources, mosquitoes exhibit plasticity in multiple feeding behaviours. If a preferred host is not found, mosquitoes are more likely to feed on sugar [32] or choose an alternative host [23], and will feed on low-sugar plant tissues when sugar-rich plants are unavailable [33]. However, despite the importance of mosquito foraging rhythms for malaria transmission and that physiological condition alters foraging rhythms in other taxa, the effect of nutritional resource availability on mosquito biting time of day is unknown.

In this study, the nutritional resources provided to adult mosquitoes were perturbed to test whether nutritional constraints cause a shift in biting time of day. A semi-field system was used to simulate scenarios in which mosquitoes have taken an initial blood meal (simulating the point at which malaria parasites would be acquired) and then resided in environments that provide varying access to further blood meals and sugar concentrations. The three scenarios represented mosquitos that: (i) were unsuccessful at acquiring a subsequent blood meal and only have access to low levels of sugar (0.5% sucrose), (ii) were unsuccessful at acquiring a subsequent blood meal but have access to high levels of sugar (10% sucrose), and (iii) successfully acquired a subsequent blood meal and have access to high levels of sugar (10% sucrose plus an additional blood meal). It was hypothesized that nutritionally deprived mosquitoes, provided with limited sugar and blood, would be more likely to host seek and shift their biting behaviour to earlier or later in the diel cycle, compared to better-resourced mosquitoes. The data was also supplemented with a preliminary field collection to investigate if biting time of day of wild-caught adult mosquitoes correlates with nutritional condition. The potential outcomes of shifts in biting time of day in response to resource availability are discussed, with emphasis on understanding residual transmission patterns, and how this may impact parasite development and transmission between hosts.

## Methods

A semi-field experiment was performed to assess if mosquito nutritional status impacts host seeking at different times of day. Wild-caught larvae were reared to adulthood, and blood fed female *Anopheles gambiae *sensu lato (*s.l.*) (thereafter called *An. gambiae*) mosquitoes from the F_2_ generation were maintained on diets that varied in nutritional resources, before they were released into an enclosed semi-field system with traps mimicking human odour. The three traps were programmed to separately capture mosquitoes biting at early (evening), classical (during the night) and late (morning) times of day. In addition, wild adult *Anopheles* mosquitoes were collected over the course of one night, to test for correlations between mosquito nutritional status and the time of day they attempted to bite.

### Study sites

For the semi-field experiment, *An. gambiae* larvae were collected from three urban sites across the Greater Accra region of Ghana (Tuba, 5° 30′ 47″ N 0° 23′ 16″ W; Teshie, 5° 35′ 0″ N, 0° 6′ 0″ W; East Legon, 5° 38′ 16.39″ N, 0° 9′ 40.33″ W) during the dry season in Dec 2023–Jan 2024 (Additional file 1, Fig. S1). Adult *An. gambiae* collections were conducted in Teshie during the dry season in February 2024. The most abundant malaria vector species in these three sites are *An. gambiae *sensu stricto (*s.s*.) and *Anopheles coluzzii*, which both have a high Human Biting Index and predominantly bite between 10 pm and 4am [34, 35]. Larvae were transported to the insectary at the Department of Medical Microbiology, University of Ghana Medical School, Accra, and raised to adults. Mosquito behavioural experiments were conducted in January–February 2024 in an enclosed semi-field system (the “MalariaSphere”, [36, 37]) at the University of Ghana campus in Legon, Accra. The average (± SEM) minimum and maximum daily temperatures during the experimental releases were 25.8 ± 0.6 °C and 34.5 ± 0.4 °C, respectively [38]. The enclosure was 5.8 × 4.2 × 2.8 m and covered in an insect-proof screen to prevent mosquito escape and/or entry from the external environment.

### Semi-field host seeking experiment

**Fig. 1:**
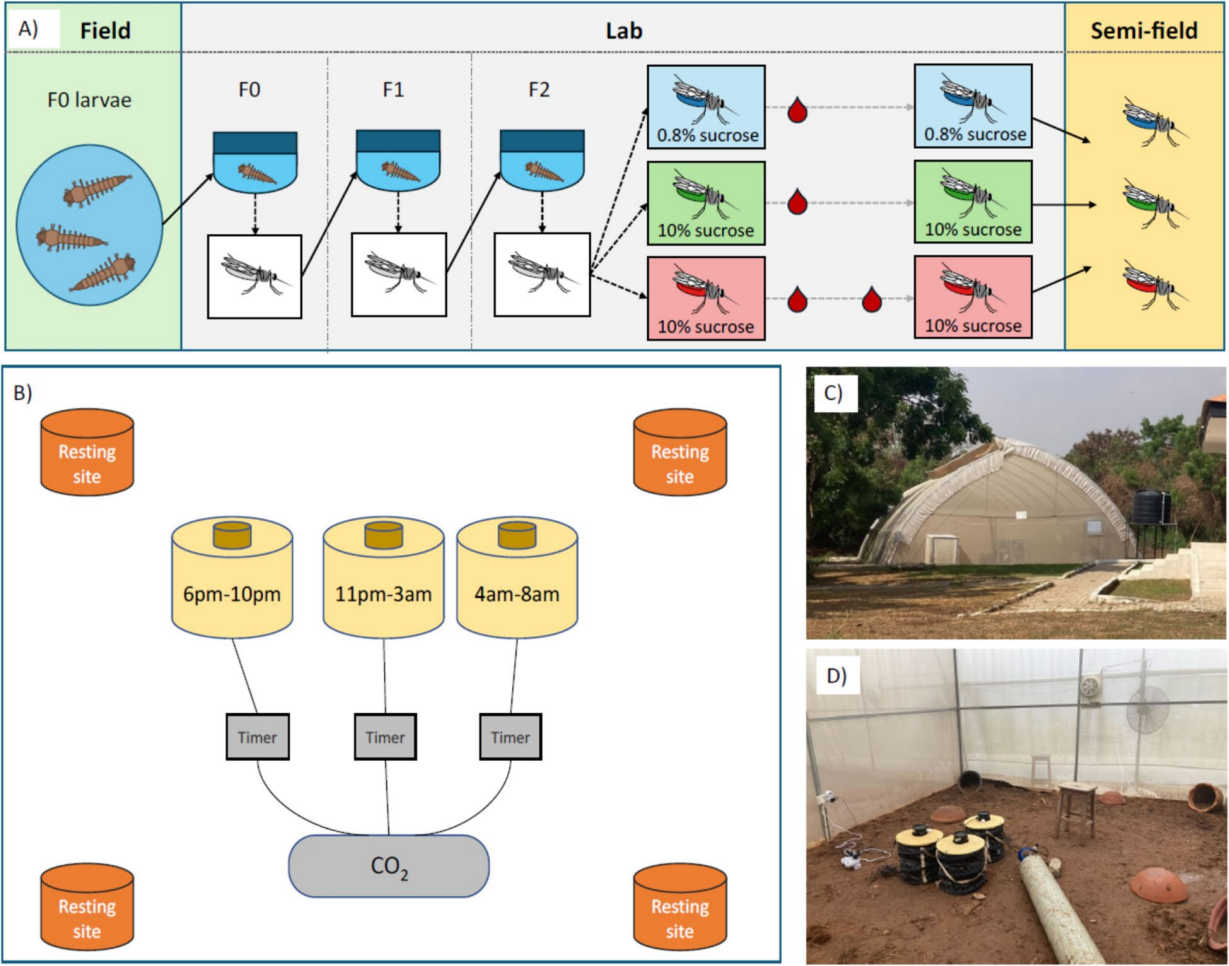
The experimental design for the semi-field behavioural experiment. Schematic diagrams of the entire experiment, from wild larvae collection to release of mosquitoes reared under the three feeding treatments into the semi-field enclosure (**A**), and the semi-field experiment set-up (**B**), featuring four resting sites for mosquitoes, and traps baited with human odour which were programmed to turn on at 6 pm–10 pm, 11 pm–3 am and 4 am–8 pm, respectively. These windows correspond to approximately ZT12-ZT16, ZT17-ZT21, and ZT22-ZT2 respectively, where Zeitgeber time (ZT) defines the hours since lights on and ZT0/24 is dawn. Each trap was linked to the CO_2_ source, using separate BG-CO_2_ timers to ensure the CO_2_ was only flowing when the corresponding trap that was switched on. Photographs show the semi-field facility (**C**) and the behavioural assay set-up (**D**)

To ensure sufficient sample sizes, semi-field experiments were conducted using the F_2_ progeny of wild-caught *An. gambiae* larvae (Fig. 1A). Collected larvae were pooled, and transferred into large tubs of water. Larvae were kept at similar densities throughout the rearing procedure and fed on TetraMin Baby fish food. The emerged adults formed the F_0_ generation, and were maintained in cages under standard rearing conditions (26 ± 2 °C, 80% relative humidity with a 12L:12D cycle), with ad libitum access to 10% sucrose solution in water. A blood meal was provided via direct-feeding [39], and soaked cotton wool in petri dishes was placed into the cages for egg laying. The resulting F_1_ generation larvae and adults were reared under the same conditions, and their eggs were used to produce the experimental F_2_ generation. On day 1 post-emergence of F_2_ adults, female mosquitoes were randomly allocated to three experimental cages (n = 100–150 per cage) and each cage was allocated to one of three feeding treatments (Fig. 1A): (i) 0.5% sucrose in water (0.5% suc), (ii) 10% sucrose in water (10% suc) or (iii) 10% sucrose in water plus an additional blood meal on day 6 post-emergence (10% suc + bm). All treatment groups were maintained on their respective sucrose solutions ad libitum from day 1 post-emergence until the host seeking experiment. In addition, to mimic mosquito life history in nature, all groups were provided with an initial blood meal on day 3 post-emergence. Mosquitoes were given the opportunity to lay eggs after all blood meals. On day 9–10 post-emergence, mosquitoes were transferred from each cage to paper cups (n = 70–100 per cup), where they were housed for 6 h with access to water only, prior to assaying biting time of day. Six batches of mosquitoes were reared to conduct a total of six semi-field system releases.

Three BG-Sentinel traps (Biogents, Germany) were placed in the semi-field enclosure, with an MB5 human-mimic lure developed to attract anopheline mosquitoes (Biogents, Germany) and a CO_2_ flow of approximately 21 g/h, to mimic human scent and breath. Each trap was programmed to turn on at either 6 pm–10 pm to capture ‘early’ biters, 11 pm–3 am to capture ‘classical’ biters, or 4 am–8 am to capture ‘late’ biters (Fig. 1B–D). These timings were chosen because mosquitoes in this region predominantly bite between 11 pm and 4 am, but biting can occur from approximately 6 pm and persist into the morning beyond 6 am [34]. Thus, these time windows also correspond to when people are typically indoors and protected by ITNs (classical), or are outdoors and unprotected (early and late) [40]. CO_2_ flow was controlled with BG-CO_2_ timers (Biogents, Germany), ensuring CO_2_ was only emitted when the respective trap was switched on.

Within the MalariaSphere, released mosquitoes could access resting sites (four clay pots around the enclosure). Water or food sources were not provided in the enclosure because this would have confounded the experimental treatment groups. Mosquitoes from each feeding treatment were colour-marked using aerosolised fluorescent powder (FTX series, Swada London), which was applied following Machani et al*.* [36]. The colour of each treatment group was rotated across sequential releases to ensure any differential impacts of colour on mosquito behaviour did not confound treatment groups. Six releases were carried out over a period of 3 weeks, with mosquitoes being released into the enclosure between 5 pm–5:30 pm, just prior to dusk at 6 pm (n = 235–300 mosquitoes per release). A period of at least 48 h between each release and checking all resting sites with a Prokopack aspirator ensured that any remaining untrapped mosquitoes in the MalariaSphere had been removed or died before the next release. Mosquitoes from the traps were collected at 8 am the day after the release, then cold-anaesthetized, and the number caught in each trap were counted.

### Wild mosquito collections

In addition to the experiment, a preliminary investigation into correlations between biting time of day and nutritional content in wild mosquitoes was conducted by collecting adult mosquitoes (n = 95) overnight between 6 pm and 7 am using the human landing catch (HLC) technique. This technique involves trained human collectors exposing their lower legs and collecting mosquitoes that land on them before they bite, and is the gold standard for investigating human exposure to disease vectors [41]. Every hour, collected mosquitoes were snap-frozen on dry ice and specimens were identified to genus level according to morphological characteristics [42], noting the number of *Anopheles* spp. mosquitoes collected per hourly bin. Individual anopheline mosquitoes were placed in 1.5 ml microcentrifuge tubes and stored on dry ice, and were transferred to a − 20 °C freezer after the overnight collection period had ended.

### Nutrition assays

Immediately prior to each semi-field release in the experiment, a subset of mosquitoes from each feeding treatment group (n = 14–19 per group, total n = 48) were collected to confirm that the resource perturbations had impacted nutritional status. Mosquito nutritional status was also quantified in individual wild-caught mosquitoes (n = 66). Mosquitoes were frozen at − 20 °C for lipid, glycogen and total sugar analyses using modified Van Handel protocols [43–45]. Firstly, a wing was removed from each mosquito to quantify body size. Wing length is a commonly used proxy for adult body size because dry weight and wing length are highly correlated [46], and is included in statistical analyses to ensure any size variation does not confound differences in nutritional content. Individual mosquitoes were lysed in 100 µl 2% sodium sulphate and 750 µl of 1:2 chloroform:methanol was added. Samples were centrifuged at 12,000 rpm for 3 min; the supernatant was used for lipid and total sugars analysis, and the precipitate was used for glycogen analysis. Lipid, glycogen and total sugars assays were conducted following Oke et al*.* [47]. For the wild-caught mosquitoes, 250 µl supernatant was used for total sugars analysis.

### Statistical analysis

Data analyses were performed using R v. 4.1.3. A linear model with wing length, mosquito release batch and their interaction as main effects was used to confirm that adult body size did not differ across the treatments (Additional file 1, Fig. S2). To analyse nutritional content (lipid, glycogen, total sugars) of individual mosquitoes, linear mixed models were conducted, with feeding treatment as a main effect and mosquito release as a random effect, accounting for any within treatment variation in body size. Binomial generalized linear mixed models (glmm), with mosquito release as a random effect to account for any between-release variation, were used to investigate how feeding treatment impacted the overall proportion of mosquitoes caught across the night, with feeding treatment as a main effect, and how feeding treatment impacted the proportion of mosquitoes caught across the different biting time of day windows, with feeding treatment, trap time and their interaction as main effects. The proportion of caught mosquitoes and relative odds ratios (OR) ± SE was estimated from models using the *emmeans* [48] package, and nutrition data was square root transformed to meet assumptions of normality and homogeneity of variance. All models were minimized using likelihood ratio tests and AICc for non-nested models, and model assumptions were confirmed using the *easystats* [49] package. To account for differences between mosquito releases, estimated marginal means ± SEM (*emmeans* package) are presented, predicted from models. Whenever the minimized model contained a significant effect of feeding treatment, post hoc pairwise comparisons were conducted using the Tukey method with the *emmeans* package.

To investigate how nutritional content between field-caught and lab-reared mosquitoes varied, linear models were used to compare between the field-caught, 0.5% suc, 10% suc and 10% suc + bm groups. A chi-square test was performed to investigate if the number of field-caught mosquitoes differed throughout the night by grouping hourly bins into ‘early’ biters (6 pm–11 pm), ‘classical’ biters (11 pm–4 am) and ‘late’ biters (4 am–7 am). Linear models were used to analyse the nutritional content (lipid, glycogen, total sugars) of individual wild-caught mosquitoes with biting time of day as an unordered factor, and to assess the correlation between wing length and nutritional content. Nutrition data were square root transformed to meet assumptions of normality and homogeneity of variance. Post hoc pairwise comparisons were conducted using the Tukey method with the *emmeans* package to compare nutritional content between field-caught and lab-reared mosquitoes.

## Results

### Resource availability perturbs mosquito nutritional status

**Fig. 2:**
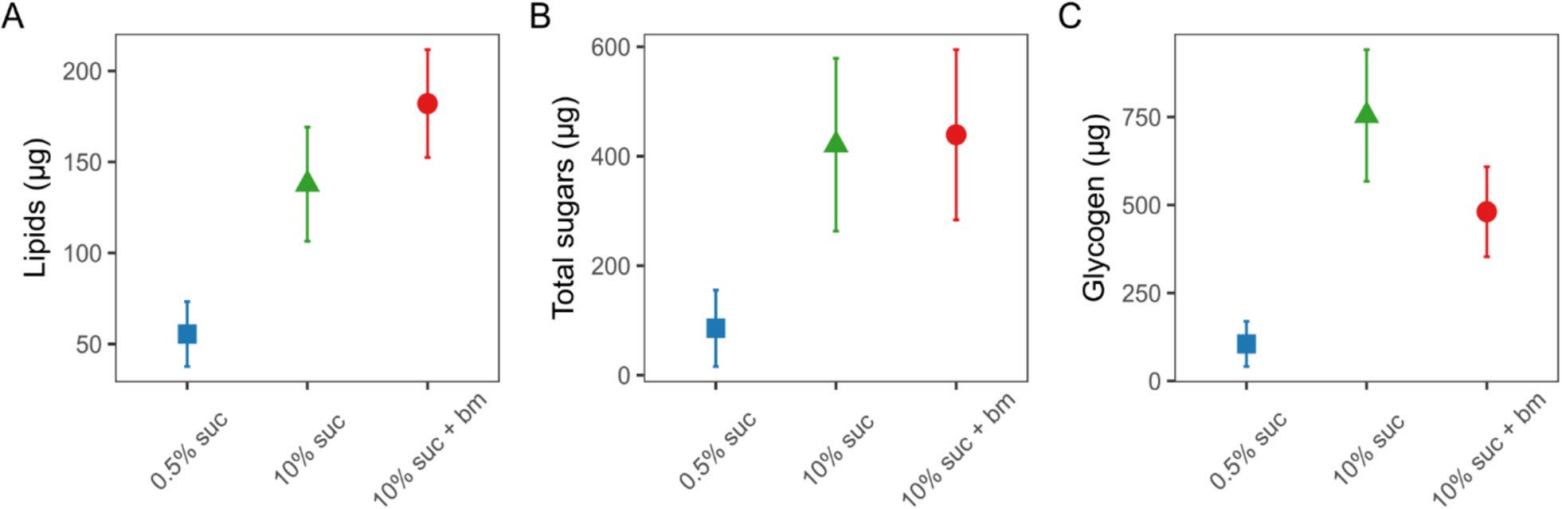
Concentrations (µg) per mosquito of lipids (**A**), total free sugars (**B**) and glycogen (**C**) in individual mosquitoes under differing feeding treatments on day 10 post-emergence. Nutritional perturbations began on day 1 post-emergence and all treatment groups received a blood meal on day 3 post-emergence (with an additional blood meal given to the 10% suc + bm group on day 6). Data presented are estimated marginal means ± SEM

Mosquitoes that fed on the lowest concentration of sucrose (0.5%) had lower nutritional reserves than mosquitoes fed 10% sucrose with or without an additional blood meal. Mosquitoes fed on 0.5% sucrose had significantly lower lipid levels (55.5 ± 17.8 µg), approximately 2.5-fold lower than those fed with 10% sucrose (138 ± 31.4 µg) and 3.3-fold lower than those fed 10% sucrose plus an additional blood meal (182 ± 29.6 µg) (χ^2^_2_ = 17.6, p < 0.001; Fig. 2A, Table 1). A similar pattern was observed for total sugars: mosquitoes fed with 0.5% sucrose had the lowest total sugar levels (85.6 ± 69.9 µg), approximately 5-fold lower than those given 10% sucrose (420 ± 158 µg) and 10% sucrose plus an additional blood meal (439 ± 156 µg) (χ^2^_2_ = 30.8, p < 0.001; Fig. 2B, Table 1). Finally, mosquitoes fed with 0.5% sucrose had the lowest glycogen levels (105 ± 64.2 µg), approximately 7-fold lower than those given 10% sucrose (754 ± 187 µg), with mosquitoes given 10% sucrose plus an additional blood meal exhibiting intermediate levels (481 ± 128 µg) (χ^2^_2_ = 21.7, p < 0.001; Fig. 2C, Table 1).

**Table 1:** Post hoc pairwise comparisons for nutritional content of mosquitoes provided with different feeding treatments

		Test statistic	p-value
Lipids	0.5% suc–10% suc	*t* = 2.75	**0.02**
	0.5% suc–10% suc + bm	*t* = − 4.54	**< 0.001**
	10% suc–10% suc + bm	*t* = − 1.16	0.48
Total sugars	0.5% suc–10% suc	*t* = 5.13	**< 0.001**
	0.5% suc–10% suc + bm	*t* = − 6.13	**< 0.001**
	10% suc–10% suc + bm	*t* = − 0.21	0.98
Glycogen	0.5% suc–10% suc	*t* = 4.66	**< 0.001**
	0.5% suc–10% suc + bm	*t* = − 3.69	**0.002**
	10% suc–10% suc + bm	*t* = 1.55	0.28

Significant p-values (< 0.05) are highlighted in bold

### Resource availability affects host seeking tendency and biting time of day

**Fig. 3:**
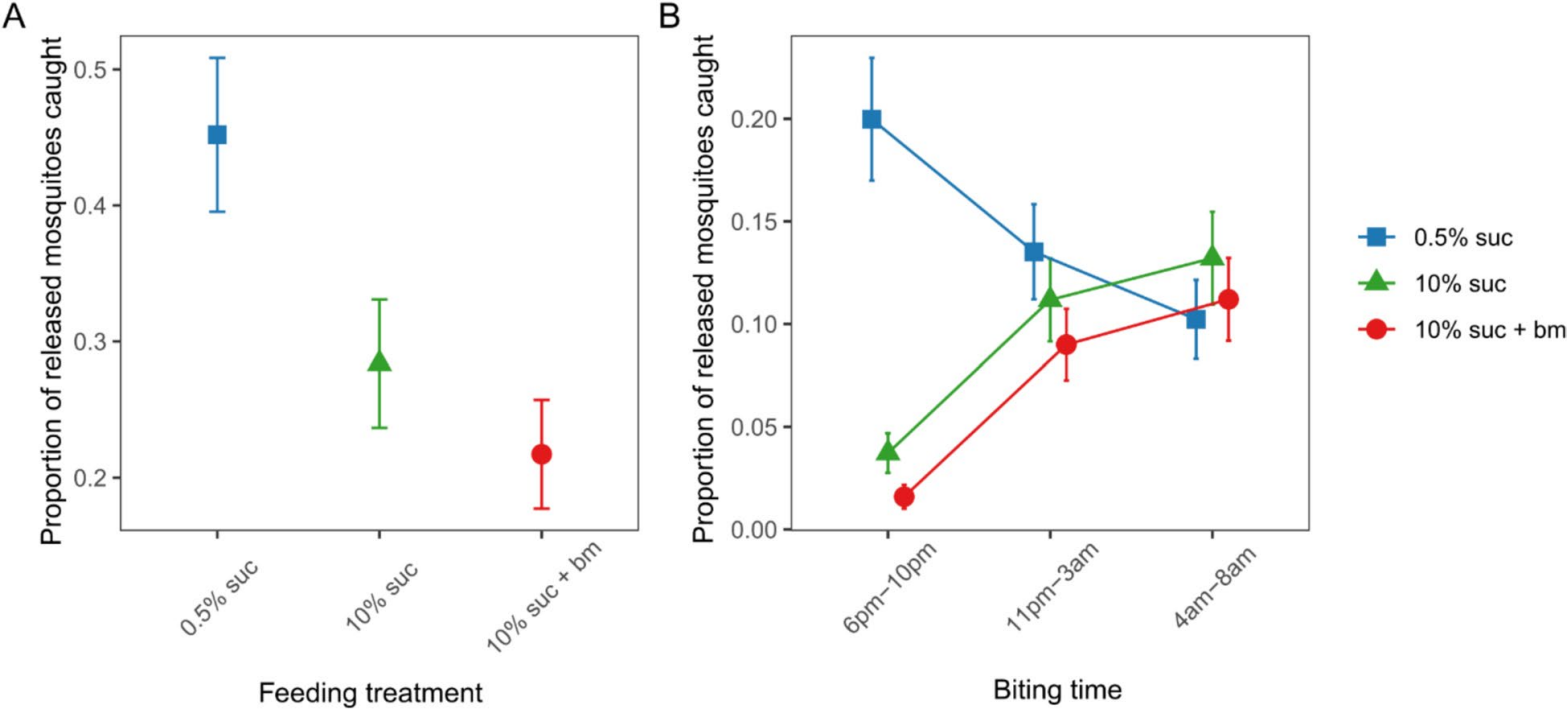
The proportion of released mosquitoes from each feeding treatment that were trapped across the entire night (**A**) and that were caught in the evening (early, 6 pm–10 pm), classical night time biting window (11 pm–3 am) and morning (late, 4 am–8 am) (**B**). Data presented are estimated marginal means ± SEM

Across all six releases and trapping times of day, the total mosquitoes trapped ranged from 21 to 49%, with a mean of 32.4% of mosquitoes caught in the traps. The likelihood of host seeking at any time of day correlated negatively with the level of nutritional resources provided (χ^2^_2_ = 65.8, p < 0.001; Fig. 3A). Specifically, mosquitoes fed 0.5% sucrose were 2-fold more likely to be trapped than those fed 10% sucrose (odds ratio (OR): 2.08 ± 0.28) and 3-fold more likely than those fed 10% sucrose plus an additional blood meal (OR: 2.97 ± 0.42). Mosquitoes fed only 10% sucrose were 1.4-fold more likely to be trapped than those fed 10% sucrose plus an additional blood meal (OR: 1.43 ± 0.21).

Nutritional resources also differentially affected the time of day of host seeking (interaction between treatment and trapping time: χ^2^_4_ = 95.4, p < 0.001; Fig. 3B). The majority of early biting mosquitoes were from the 0.5% sucrose group, whereas mosquitoes with more resources were more likely to be caught at classic and late times of day. Specifically, mosquitoes fed 0.5% sucrose were approximately 5- and 10-fold more likely to be caught in the early biting window (20 ± 2.9%) than those fed with 10% sucrose (3.7 ± 1.0%) and 10% sucrose plus an additional blood meal (1.6 ± 0.6%), respectively. At the classical and late trapping times of day, the proportion of mosquitoes fed 0.5% sucrose caught was similar to mosquitoes fed 10% sucrose with or without an additional blood meal. Mosquitoes fed 10% sucrose with or without an additional blood meal followed similar temporal patterns across all trapping times of day, with approximately 4-fold more of these mosquitoes being trapped during the classical (10% sucrose: 11 ± 2.0%, 10% sucrose + bm: 9.0 ± 1.7%) and late biting windows (10% sucrose: 13 ± 2.3%, 10% sucrose + bm: 11 ± 2.0%) than during the early biting window (10% sucrose: 3.7 ± 1.0%, 10% sucrose + bm: 1.6 ± 0.6%).

### Biting time distribution and nutritional status of wild host-seeking mosquitoes

**Fig. 4:**
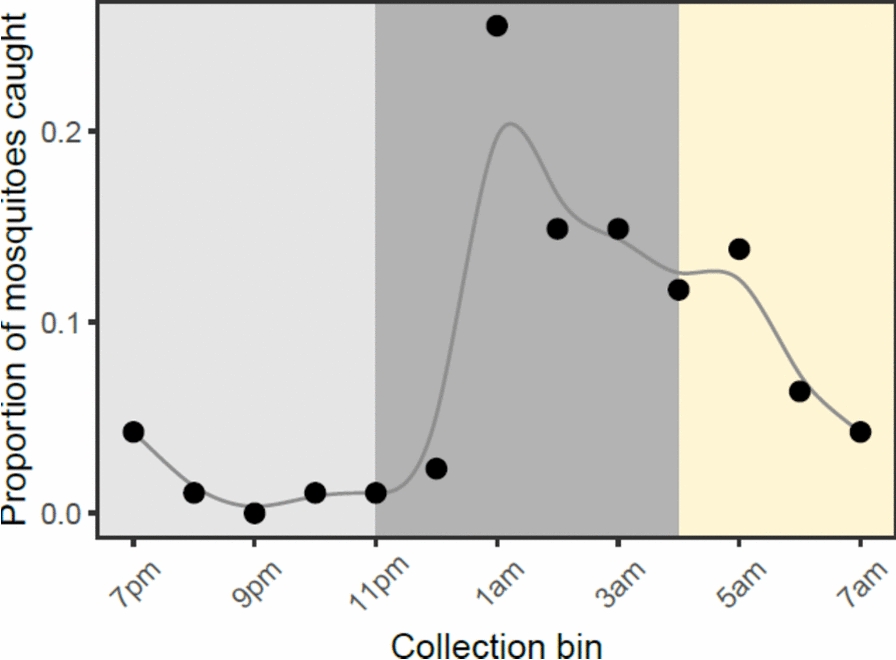
The proportion of wild adult *An. gambiae* mosquitoes caught by HLC collections across hourly bins between 6 pm and 7 am. Early, classical, and late bins are indicated by shaded regions from left to right. To highlight how the pattern changes over the course of the night, data points are connected by an X-spline

A preliminary collection of wild host-seeking *An. gambiae *mosquitoes was conducted to assess whether mosquito nutritional status correlates with biting time of day. A total of 95 wild host seeking mosquitoes were caught during the overnight collection, with a peak in biting between 12 am–1 am. Hourly bins were grouped into ‘early’ biters (6 pm–11 pm), ‘classical’ biters (11 pm–4 am) and ‘late’ biters (4 am–7 am), and there were differences in number of mosquitoes caught across these windows. Specifically, 68% of mosquitoes were caught during the classical biting window, compared to 7% and 24% caught during the evening and morning windows respectively, confirming that this population predominantly bite during the ‘classical’ time of day (χ^2^_2_ = 46.1, p < 0.001; Fig. 4).

Although wild mosquitoes varied in their nutritional content, there were no differences between lipid (F_9,56_ = 0.75, p = 0.66), sugars (F_9,56_ = 1.06, p = 0.40) or glycogen (F_9,56_ = 0.40, p = 0.93) contents across hourly bins (Additional file 1, Fig S3). However, wild host-seeking mosquitoes had similar nutritional reserves to the experimental mosquitoes fed a 0.5% sucrose diet (estimated marginal means for field-caught mosquito lipids: 39.7 ± 5.5 μg, sugars: 65.2 ± 13 μg, and glycogen: 51.4 ± 11 μg), but had significantly lower reserves than mosquitoes fed 10% sucrose and 10% sucrose plus an additional blood meal (lipid: F_3,110_ = 24.9, p < 0.001; sugars: F_3,110_ = 26.0, p < 0.001; glycogen: F_3,110_ = 58.2, p < 0.001; Table 2).

**Table 2:** Post hoc pairwise comparisons of the nutritional content of field-caught mosquitoes versus. lab-reared mosquitoes on different feeding treatments

		Test statistic	p-value
Lipids	Field–0.5% suc	*t* = − 1.26	0.59
	Field–10% suc	*t* = − 4.83	**< 0.001**
	Field–10% suc + bm	*t* = − 7.93	**< 0.001**
Total sugars	Field–0.5% suc	*t* = − 1.14	0.67
	Field–10% suc	*t* = − 4.96	**< 0.001**
	Field–10% suc + bm	*t* = − 8.07	**< 0.001**
Glycogen	Field–0.5% suc	*t* = − 1.97	0.21
	Field–10% suc	*t* = − 11.3	** < 0.001**
	Field–10% suc + bm	*t* = − 8.87	**< 0.001**

Significant p-values (< 0.05) are highlighted in bold

## Discussion

By altering sugar provision and access to blood meals, and using traps that mimic human breath and scent, this study demonstrates that resource availability modulates the time of day that *An. gambiae* mosquitoes in Ghana search for a blood meal. Mosquitoes on a low sucrose diet had significantly lower levels of lipids, glycogen and sugar, and were 5–10-fold more likely to host seek in the evening (early) than mosquitoes on a high sucrose diet with or without a blood meal (Figs. 2, 3B). In contrast, better-resourced mosquitoes were most likely to host seek during the classical biting time window and into the morning (Figs. 2, 3B). The results also show that as resources increased, the overall tendency to host seek decreased; mosquitoes on a low sucrose diet were 2-fold more likely to be caught compared to mosquitoes given a higher concentration of sucrose, and 3-fold more likely than those with access to 10% sucrose and an additional blood meal (Fig. 3A). Finally, to probe the ecological relevance of the experimental results, a trapping session of wild host-seeking *An. gambiae* mosquitoes was conducted. While the nutritional content of wild mosquitoes varied, levels were similar to the profiles of the low-resourced experimental mosquitoes fed 0.5% sucrose. Given that mosquito flight during host-seeking is energetically consuming [50], this suggests that the experimental feeding treatments resulted in mosquitoes with nutritional contents within an ecologically realistic range.

Foraging at unusual times of day can be detrimental because it exposes organisms to rhythmic environmental risks, such as the active phase of predators and suboptimal temperature/humidity, and misalignment between metabolic and other circadian rhythms may impair digestion and metabolism [1]. However, under nutritional stress, finding food becomes more critical for survival, outweighing the environmental risks and physiological costs [18, 21]. For example, a poorly-resourced mosquito may begin host seeking earlier in the night because their lack of energy reserves reduce the chance of surviving until later into the night (or the morning, depending on whether ITNs limit access to hosts at night). The results of this study are consistent with this hypothesis because poorly-resourced mosquitoes with low nutritional reserves are more likely to host seek, predominantly in the early evening when temperatures are higher and humidity is lower, and they risk being killed because human hosts are alert. Although survival was not quantified, live mosquitoes fed on a 0.5% sucrose diet were not encountered whilst clearing the semi-field system the day after each release, but live mosquitoes fed on higher sucrose solutions and/or an additional blood meal were regularly encountered. This indicates that mosquitoes fed 0.5% sucrose were low-resourced and did not have sufficient reserves to survive until morning. Overall, these findings suggest that early biting could be an adaptive plastic temporal shift to maximize fitness in sugar-poor regions and where hosts are not easily accessible due to high ITN use.

Better-resourced mosquitoes on high sucrose diets (with or without an additional blood meal) had higher nutritional reserves and waited until the classical time window to host seek, in alignment with their circadian physiology and when environmental risks are likely to be lower, which also supports the proposed hypothesis. However, better-resourced mosquitoes were just as likely as poorly-resourced mosquitoes to be trapped later in the morning. Such late biting by well-fed mosquitoes was unexpected but may be due to the lack of water/food sources provided in the semi-field enclosures, forcing untrapped mosquitoes to host seek to mitigate dehydration caused by the rise in temperature and the reduction in humidity in the morning [51, 52]. Alternatively, well-resourced mosquitoes which had failed to locate a host (*i.e.* had not entered a trap) during the night may have become resource-depleted because flight is energetically demanding [50], leading to an increased propensity to continue host seeking because they do not have sufficient energy to survive until the subsequent night. Regardless of the explanation(s), this study reveals that adult nutritional condition drives variation in biting time of day.

The semi-field system allowed mosquitoes to experience ecologically realistic temperature and humidity fluctuations and provided sufficient flight space, but excluded other ecological factors, including predation and access to alternative food sources. Thus, to fully investigate how resource availability impacts biting time, future studies should consider adding plants of varying sugar contents and alternative blood hosts into semi-field systems. However, these omissions were necessary in this experiment to isolate the effect of adult nutritional status on biting behaviour. To probe the ecological relevance of the experimental results, a preliminary trapping session of wild host-seeking *An. gambiae* mosquitoes was also conducted. Wild mosquitoes had similar nutritional profiles to the low-resourced mosquitoes fed on 0.5% sucrose, suggesting that the experimental perturbations represented natural conditions. The low sample size for wild caught mosquitoes precludes comparing nutritional reserves between host-seeking times of day but does suggest that adult nutritional content does not correlate with body size (a product of larval condition) (Additional file 1, Fig. S4). Thus, catching lowly resourced mosquitoes at the time of host seeking is consistent with the experimental results revealing that adult nutrition influences biting rhythms. Further field studies are necessary to build on these findings, including sampling indoor and outdoor host-seeking mosquitoes across multiple sites throughout a 24-h period, examining their nutritional status, and investigating when mosquitoes become active.

Like previous studies across Africa [3, 35, 53], a large proportion of wild mosquitoes (approximately 20%) were caught host-seeking during the morning when humans are unprotected by ITNs. This pattern of ‘late’ biting has become more pronounced since the widespread use of ITNs. Because biting time shifts have a genetic component [16], and universal coverage of ITNs is approximately 75% in Ghana [54], late biting could be an evasion strategy evolving in the mosquito population where the larval collections for the experiment were carried out, rather than an entirely plastic behavioural change. Further work is needed to ascertain the extent to which biting time of day is genetically determined and/or due to behavioural plasticity, because plasticity itself evolves and can facilitate or constrain evolution that genetically segregates mosquito populations into early, classical or late biters. Modelling suggests that early biting is more likely to evolve in sugar-poor environments than sugar-rich environments, for example, due to differences in the abundance and diversity of plants [24]. However, in areas of fluctuating resource availability or ITN coverage, plastic genotypes that can switch between early, classical, or late biting depending on their environmental conditions should have higher fitness [17].

Both the semi-field experiment and preliminary field data reveal significant variation in biting time of day, and intuition suggests that early biting by poorly-resourced mosquitoes could sustain malaria transmission. However, the overall impact of shifts in mosquito biting time of day for malaria transmission is difficult to predict, because of complex and potentially opposing effects on both mosquito and parasite fitness. While early biting causes greater contact between vectors and hosts in regions with high ITN coverage, malaria parasite development is less productive in low-resourced mosquitoes, and mosquito survival is reduced [47]. Thus, the poor nutritional state of early biters could curtail onward transmission through lower parasite numbers [55] and because vectors may not survive long enough for malaria parasites to complete their development [56, 57]. In addition, parasite establishment in mosquitoes is impacted by daily temperature rhythms [58] and parasite infectivity and mosquito susceptibility vary between day and night [1, 59, 60]. Disruption to foraging rhythms also reduces fitness in other insects [61], and in mosquitoes could lead to mismatch between metabolic processes and blood digestion [62]. The net outcome for malaria transmission of these myriad and potentially synergistic and antagonistic effects of biting time of day is challenging to predict. However, recognizing that transmission is time of day dependent is necessary for accurately predicting epidemiology, the risk of clinical malaria cases, and the trajectory of parasite evolution.

## Conclusions

The results of this study suggest that plasticity in biting time of day, driven by variation in adult mosquito nutritional condition, may be an underappreciated contributor to residual malaria transmission. In particular, nutritional stress in mosquito populations may lead to shifts in biting behaviour, reducing ITN efficacy. This highlights that mosquito nutrition and its influence on the timing of biting behaviour should be considered when implementing and assessing vector control strategies, especially those which target mosquito foraging behaviour, such as ITNs and sugar-baited traps. More broadly, investigating the drivers of variation in biting time of day and the overall impact on pathogen transmission is critical for assessing and improving the efficacy of current and future vector control tools. This is especially important for malaria, where up to 30% of biting is reported to occur when humans are unprotected by ITNs and is predicted to lead to millions of additional clinical cases per year [3, 10]. However, these findings are likely to apply to other vector-borne diseases that rely on vector foraging rhythms for between-host transmission.

## Supplementary Information

Additional file 1. Figure S1 is a map of larval and adult collection sites, Figure S2 is the wing length of lab-reared mosquitoes under different feeding treatments, Figure S3 provides information about the nutritional contents of wild adult mosquitoes collected at different times of day, and Figure S4 is the correlation between wing length and nutritional content in wild adult mosquitoes.

## Data Availability

The datasets supporting the conclusions of this article are available in the Edinburgh DataShare repository and can be accessed with [10.7488/ds/8017].

## References

[1] Rund SSC, O’Donnell AJ, Gentile JE, Reece SE. Daily rhythms in mosquitoes and their consequences for malaria transmission. Insects. 2016;7:14. 27089370 10.3390/insects7020014 PMC4931426

[2] Westwood ML, O’Donnell AJ, de Bekker C, Lively CM, Zuk M, Reece SE. The evolutionary ecology of circadian rhythms in infection. Nat Ecol Evol. 2019;3:552–60. 30886375 10.1038/s41559-019-0831-4 PMC7614806

[3] Sangbakembi-Ngounou C, Costantini C, Longo-Pendy NM, Ngoagouni C, Akone-Ella O, Rahola N. Diurnal biting of malaria mosquitoes in the Central African Republic indicates residual transmission may be “out of control.” Proc Natl Acad Sci USA. 2022;119:e2104282119. 35576470 10.1073/pnas.2104282119 PMC9173762

[4] Thomsen EK, Koimbu G, Pulford J, Jamea-Maiasa S, Ura Y, Keven JB, et al. Mosquito behavior change after distribution of bednets results in decreased protection against malaria exposure. J Infect Dis. 2017;215:790–7. 28007921 10.1093/infdis/jiw615 PMC5388271

[5] Moiroux N, Gomez MB, Pennetier C, Elanga E, Djènontin A, Chandre F, et al. Changes in *Anopheles funestus* biting behavior following universal coverage of long-lasting insecticidal nets in Benin. J Infect Dis. 2012;206:1622–9. 22966127 10.1093/infdis/jis565

[6] Oke CE, Ingham VA, Walling CA, Reece SE. Vector control: agents of selection on malaria parasites? Trends Parasitol. 2022;38:890–903. 35981937 10.1016/j.pt.2022.07.006

[7] WHO. World Malaria Report 2023. Geneva: World Health Organisation; 2023.

[8] Bhatt S, Weiss DJ, Cameron E, Bisanzio D, Mappin B, Dalrymple U, et al. The effect of malaria control on *Plasmodium falciparum* in Africa between 2000 and 2015. Nature. 2015;526:207–11. 26375008 10.1038/nature15535 PMC4820050

[9] Huijben S, Paaijmans KP. Putting evolution in elimination: winning our ongoing battle with evolving malaria mosquitoes and parasites. Evol Appl. 2018;11:415–30. 29636796 10.1111/eva.12530 PMC5891050

[10] Sherrard-Smith E, Skarp JE, Beale AD, Fornadel C, Norris LC, Moore SJ, et al. Mosquito feeding behavior and how it influences residual malaria transmission across Africa. Proc Natl Acad Sci USA. 2019;116:15086–96. 31285346 10.1073/pnas.1820646116 PMC6660788

[11] Russell TL, Beebe NW, Cooper RD, Lobo NF, Burkot TR. Successful malaria elimination strategies require interventions that target changing vector behaviours. Malar J. 2013;12:56. 23388506 10.1186/1475-2875-12-56 PMC3570334

[12] Sougoufara S, Diédhiou SM, Doucouré S, Diagne N, Sembène PM, Harry M, et al. Biting by *Anopheles funestus* in broad daylight after use of long-lasting insecticidal nets: a new challenge to malaria elimination. Malar J. 2014;13:125. 24678587 10.1186/1475-2875-13-125 PMC3973838

[13] Yohannes M, Boelee E. Early biting rhythm in the afro-tropical vector of malaria, *Anopheles arabiensis*, and challenges for its control in Ethiopia. Med Vet Entomol. 2012;26:103–5. 21410494 10.1111/j.1365-2915.2011.00955.x

[14] Cooke MK, Kahindi SC, Oriango RM, Owaga C, Ayoma E, Mabuka D, et al. “A bite before bed”: exposure to malaria vectors outside the times of net use in the highlands of western Kenya. Malar J. 2015;14:259. 26109384 10.1186/s12936-015-0766-4 PMC4479228

[15] Carrasco D, Lefèvre T, Moiroux N, Pennetier C, Chandre F, Cohuet A. Behavioural adaptations of mosquito vectors to insecticide control. Curr Opin Insect Sci. 2019;34:48–54. 31247417 10.1016/j.cois.2019.03.005

[16] Govella NJ, Johnson PCD, Killeen GF, Ferguson HM. Heritability of biting time behaviours in the major African malaria vector *Anopheles arabiensis*. Malar J. 2023;22:228. 37587487 10.1186/s12936-023-04671-7 PMC10433675

[17] Pigliucci M. Evolution of phenotypic plasticity: where are we going now? Trends Ecol Evol. 2005;20:481–6. 16701424 10.1016/j.tree.2005.06.001

[18] Van Der Veen DR, Riede SJ, Heideman PD, Hau M, Van Der Vinne V, Hut RA. Flexible clock systems: adjusting the temporal programme. Phil Trans R Soc Lond B Biol Sci. 2017;372:20160254. 28993498 10.1098/rstb.2016.0254 PMC5647281

[19] Van Der Vinne V, Riede SJ, Gorter JA, Eijer WG, Sellix MT, Menaker M, et al. Cold and hunger induce diurnality in a nocturnal mammal. Proc Natl Acad Sci U S A. 2014;111:15256–60. 25288753 10.1073/pnas.1413135111 PMC4210334

[20] Van Der Vinne V, Gorter JA, Riede SJ, Hut RA. Diurnality as an energy-saving strategy: energetic consequences of temporal niche switching in small mammals. J Exp Biol. 2015;218:2585–93. 26290592 10.1242/jeb.119354

[21] Van Der Vinne V, Tachinardi P, Riede SJ, Akkerman J, Scheepe J, Daan S, et al. Maximising survival by shifting the daily timing of activity. Ecol Lett. 2019;22:2097–102. 31617283 10.1111/ele.13404 PMC6899458

[22] Sougoufara S, Harry M, Doucouré S, Sembène PM, Sokhna C. Shift in species composition in the *Anopheles gambiae* complex after implementation of long-lasting insecticidal nets in Dielmo. Senegal Med Vet Entomol. 2016;30:365–8. 27058993 10.1111/mve.12171

[23] Kreppel KS, Viana M, Main BJ, Johnson PCD, Govella NJ, Lee Y, et al. Emergence of behavioural avoidance strategies of malaria vectors in areas of high LLIN coverage in Tanzania. Sci Rep. 2020;10:14527. 32883976 10.1038/s41598-020-71187-4 PMC7471940

[24] Stone C, Chitnis N, Gross K. Environmental influences on mosquito foraging and integrated vector management can delay the evolution of behavioral resistance. Evol Appl. 2016;9:502–17. 26989441 10.1111/eva.12354 PMC4778105

[25] Barredo E, Degennaro M. Not just from blood: mosquito nutrient acquisition from nectar sources. Trends Parasitol. 2020;36:473–84. 32298634 10.1016/j.pt.2020.02.003

[26] Rund SSC, Bonar NA, Champion MM, Ghazi JP, Houk CM, Leming MT, et al. Daily rhythms in antennal protein and olfactory sensitivity in the malaria mosquito *Anopheles gambiae*. Sci Rep. 2013;3:2494. 23986098 10.1038/srep02494 PMC3756343

[27] Mitchell SN, Catteruccia F. Anopheline reproductive biology: impacts on vectorial capacity and potential avenues for malaria control. Cold Spring Harb Perspect Med. 2017;7:14. 10.1101/cshperspect.a025593 PMC5710097 28389513

[28] Foster WA. Behavioural ecology of plant-mosquito relations. In: Ignell R, Lazzari CR, Lorenzo MG, Hill SR, editors. Sensory ecology of disease vectors. The Netherlands: Wageningen Academic Publishers; 2022. 37285450

[29] Ebrahimi B, Jackson BT, Guseman JL, Przybylowicz CM, Stone CM, Foster WA. Alteration of plant species assemblages can decrease the transmission potential of malaria mosquitoes. J Appl Ecol. 2018;55:841–51. 29551835 10.1111/1365-2664.13001 PMC5849257

[30] Gu W, Müller G, Schlein Y, Novak RJ, Beier JC. Natural plant sugar sources of *Anopheles* mosquitoes strongly impact malaria transmission potential. PLoS ONE. 2011;6:e15996. 21283732 10.1371/journal.pone.0015996 PMC3024498

[31] Killeen GF, Smith TA, Ferguson HM, Mshinda H, Abdulla S, Lengeler C, et al. Preventing childhood malaria in Africa by protecting adults from mosquitoes with insecticide-treated nets. PLoS Med. 2007;4:1246–58. 10.1371/journal.pmed.0040229 PMC1904465 17608562

[32] Gary RE, Foster WA. Diel timing and frequency of sugar feeding in the mosquito *Anopheles gambiae*, depending on sex, gonotrophic state and resource availability. Med Vet Entomol. 2006;20:308–16. 17044882 10.1111/j.1365-2915.2006.00638.x

[33] Müller G, Schlein Y. Plant tissues: the frugal diet of mosquitoes in adverse conditions. Med Vet Entomol. 2005;19:413–22. 16336306 10.1111/j.1365-2915.2005.00590.x

[34] Sabtiu ARM, Sraku IK, Mfum CA, Akuamoah-Boateng Y, Doe RT, Boadu EN, et al. Malaria transmission risk in the city of Accra, Ghana: vector behavior and distribution. Res Sq. 2025. 10.21203/rs.3.rs-6008722/v1.40470219

[35] Akuoko OK, Dhikrullahi SB, Hinne IA, Mohammed AR, Mfum Owusu-Asenso C, Coleman S, et al. Biting behaviour and the spatio-temporal dynamics of malaria vectors in different ecological zones in Ghana. Parasit Vectors. 2024;17:16. 38195546 10.1186/s13071-023-06065-9 PMC10775458

[36] Machani MG, Ochomo E, Amimo F, Mukabana WR, Githeko AK, Yan G, et al. Behavioral responses of pyrethroid resistant and susceptible *Anopheles gambiae* mosquitoes to insecticide treated bed net. PLoS ONE. 2022;17:e0266420. 35390050 10.1371/journal.pone.0266420 PMC8989192

[37] Osoro JK, Machani MG, Ochomo E, Wanjala C, Omukunda E, Githeko AK, et al. Insecticide resistant *Anopheles gambiae* have enhanced longevity but reduced reproductive fitness and a longer first gonotrophic cycle. Sci Rep. 2022;12:8646. 35606505 10.1038/s41598-022-12753-w PMC9126871

[38] Valer GB. Ogimet. 2024. https://www.ogimet.com/cgi-bin/gsynres?lang=en&ind=65472&ndays=21&ano=2024&mes=02&day=10&hora=11&ord

[39] Harrington LC, Foy BD, Bangs MJ. Considerations for human blood-feeding and arthropod exposure in vector biology research: an essential tool for investigations and disease control. Vector Borne Zoonotic Dis. 2020;20:807–16. 32905735 10.1089/vbz.2020.2620 PMC7698847

[40] Nzioki I, Machani MG, Onyango SA, Kabui KK, Githeko AK, Ochomo E, et al. Current observations on shifts in malaria vector biting behavior and changing vulnerability to malaria transmission in contrasting ecosystems in Western Kenya. Parasit Vectors. 2023;16:376. 37864217 10.1186/s13071-023-05944-5 PMC10590029

[41] Gimnig JE, Walker ED, Otieno P, Kosgei JK, Olang GB, Ombok MO, et al. Incidence of malaria among mosquito collectors conducting human landing catches in Western Kenya. Am J Trop Med Hyg. 2013;88:301–8. 23249685 10.4269/ajtmh.2012.12-0209 PMC3583321

[42] Gillies MT, Coetzee M. A supplement to the Anophelinae of Africa South of the Sahara. Publ S Afr Inst Med Res. 1987;55:1–143.

[43] Van Handel E, Day JF. Assay of lipids, glycogen and sugars in individual mosquitoes: correlations with wing length in field-collected *Aedes vexans*. J Am Mosq Control Assoc. 1988;4:549–50. 3225576

[44] Van Handel E. Rapid determination of glycogen and sugars in mosquitoes. J Am Mosq Control Assoc. 1985;1:299–301. 2906671

[45] Van Handel E. Rapid determination of total lipids in mosquitoes. J Am Mosq Control Assoc. 1985;1:1302–4. 2906672

[46] Lehmann T, Dalton R, Kim EH, Dahl E, Diabate A, Dabire R, et al. Genetic contribution to variation in larval development time, adult size, and longevity of starved adults of *Anopheles gambiae*. Infect Genet Evol. 2006;6:410–6. 16524787 10.1016/j.meegid.2006.01.007

[47] Oke CE, O’Donnell AJ, Schneider P, Reece SE. Plasticity in malaria parasite development: mosquito resources influence vector-to-host transmission potential. Front Malar. 2024;2:1481816.

[48] Lenth RV. emmeans: Estimated Marginal Means, aka Least-Squares Means. 2023. https://cran.r-project.org/package=emmeans

[49] Makowski D, Ben-Shachar M, Lüdecke D. The {easystats} collection of R packages. 2020. https://easystats.github.io/easystats/

[50] Kaufmann C, Briegel H. Flight performance of the malaria vectors *Anopheles gambiae* and *Anopheles atroparvus*. J Vector Ecol. 2004;29:140–53. 15266751

[51] Hagan RW, Didion EM, Rosselot AE, Holmes CJ, Siler SC, Rosendale AJ, et al. Dehydration prompts increased activity and blood feeding by mosquitoes. Sci Rep. 2018;8:6804. 29717151 10.1038/s41598-018-24893-z PMC5931509

[52] Lin S, Senapati B, Tsao CH. Neural basis of hunger-driven behaviour in *Drosophila*. Open Biol. 2019;9:180259. 30914005 10.1098/rsob.180259 PMC6451361

[53] Odero JI, Abong’o B, Moshi V, Ekodir S, Harvey SA, Ochomo E, et al. Early morning anopheline mosquito biting, a potential driver of malaria transmission in Busia County, western Kenya. Malar J. 2024;23:66. 38438933 10.1186/s12936-024-04893-3 PMC10910777

[54] Afagbedzi SK, Alhassan Y, Kenu E, Malm K, Bandoh DAB, Peprah NY, et al. Universal coverage and utilization of free long-lasting insecticidal nets for malaria prevention in Ghana: a cross-sectional study. Front Public Health. 2023;11:1140604. 37304125 10.3389/fpubh.2023.1140604 PMC10248059

[55] Aleshnick M, Ganusov VV, Nasir G, Yenokyan G, Sinnis P. Experimental determination of the force of malaria infection reveals a non-linear relationship to mosquito sporozoite loads. PLoS Pathog. 2020;16:e1008181. 32453765 10.1371/journal.ppat.1008181 PMC7295235

[56] Ohm JR, Baldini F, Barreaux P, Lefevre T, Lynch PA, Suh E, et al. Rethinking the extrinsic incubation period of malaria parasites. Parasit Vectors. 2018;11:178. 29530073 10.1186/s13071-018-2761-4 PMC5848458

[57] Shaw WR, Holmdahl I, Itoe M, Werling K, Marquette M, Paton D, et al. Multiple blood feeding in mosquitoes shortens the *Plasmodium falciparum* incubation period and increases malaria transmission potential. PLoS Pathog. 2020;16:e1009131. 33382824 10.1371/journal.ppat.1009131 PMC7774842

[58] Suh E, Grossman MK, Waite JL, Dennington NL, Sherrard-Smith E, Churcher TS, et al. The influence of feeding behaviour and temperature on the capacity of mosquitoes to transmit malaria. Nat Ecol Evol. 2020;4:940–51. 32367033 10.1038/s41559-020-1182-x PMC7334094

[59] Pigeault R, Caudron Q, Nicot A, Rivero A, Gandon S. Timing malaria transmission with mosquito fluctuations. Evol Lett. 2018;2:378–89. 30283689 10.1002/evl3.61 PMC6122125

[60] Schneider P, Rund SSC, Smith NL, Prior KF, O’Donnell AJ, Reece SE. Adaptive periodicity in the infectivity of malaria gametocytes to mosquitoes. Proc Biol Sci. 2018;285:20181876. 30282657 10.1098/rspb.2018.1876 PMC6191691

[61] Gill S, Le HD, Melkani GC, Panda S. Time-restricted feeding attenuates age-related cardiac decline in *Drosophila*. Science. 2015;347:1265–9. 25766238 10.1126/science.1256682 PMC4578815

[62] Rund SSC, Hou TY, Ward SM, Collins FH, Duffield GE. Genome-wide profiling of diel and circadian gene expression in the malaria vector *Anopheles gambiae*. Proc Natl Acad Sci U S A. 2011;108:E421–30. 21715657 10.1073/pnas.1100584108 PMC3156198

